# Wheat grain width: a clue for re-exploring visual indicators of grain weight

**DOI:** 10.1186/s13007-022-00891-1

**Published:** 2022-05-03

**Authors:** Abbas Haghshenas, Yahya Emam, Saeid Jafarizadeh

**Affiliations:** 1 Department of Plant Production and Genetics, Shiraz University, Shiraz, Iran; 2 Vision Lab, Electrical and Computer Engineering School, Shiraz University, Shiraz, Iran

**Keywords:** Cultivar mixture, Grain shape, Image processing, Phenotyping

## Abstract

**Background:**

Mean grain weight (MGW) is among the most frequently measured parameters in wheat breeding and physiology. Although in the recent decades, various wheat grain analyses (e.g. counting, and determining the size, color, or shape features) have been facilitated, thanks to the automated image processing systems, MGW estimations have been limited to using few number of image-derived indices; i.e. mainly the linear or power models developed based on the projected area (*Area*). Following a preliminary observation which indicated the potential of grain width in improving the predictions, the present study was conducted to explore more efficient indices for increasing the precision of image-based MGW estimations. For this purpose, an image archive of the grains was processed, which were harvested from a 2-year field experiment carried out with 3 replicates under two irrigation conditions and included 15 cultivar mixture treatments (so the archive was consisted of 180 images including more than 72,000 grains).

**Results:**

It was observed that among the more than 30 evaluated indices of grain size and shape, indicators of grain width (i.e. *Minor* & *MinFeret*) along with 8 other empirical indices had a higher correlation with MGW, compared with *Area*. The most precise MGW predictions were obtained using the *Area* × *Circularity*, *Perimeter* × *Circularity*, and *Area/Perimeter* indices. Furthermore, it was found that (i) grain width and the *Area/Perimeter* ratio were the common factors in the structure of the superior predictive indices; and (ii) the superior indices had the highest correlation with grain width, rather than with their mathematical components. Moreover, comparative efficiency of the superior indices almost remained stable across the 4 environmental conditions. Eventually, using the selected indices, ten simple linear models were developed and validated for MGW prediction, which indicated a relatively higher precision than the current *Area*-based models. The considerable effect of enhancing image resolution on the precision of the models has been also evidenced.

**Conclusions:**

It is expected that the findings of the present study, along with the simple predictive linear models developed and validated using new image-derived indices, could improve the precision of the image-based MGW estimations, and consequently facilitate wheat breeding and physiological assessments.

**Supplementary Information:**

The online version contains supplementary material available at 10.1186/s13007-022-00891-1.

## Background

Although number of grains per unit of area is known to be the most important component of wheat yield [1, 2], grain weight and its related features (e.g. size and shape) are still under consideration of researchers for improving the yield capacity (e.g. see [3–6]). Accordingly, wheat grain has been well-explored visually in the recent decades, either using uncomplicated methods and 2D indices [4, 7–11] or employing more complex techniques of 3D reconstruction [12–14]. In spite of the fact that the current advanced technology of X-ray computed tomography has provided almost any kind of data required for geometric assessment of wheat grain e.g. see [15–18], utilizing this approach is time consuming, expensive, and limited to comparatively less available specific CT scanners. Moreover, reconstruction and analysis of 3D structures requires a more sophisticated level of image processing. In contrast, 2D analysis of grins based on common digital images, is low-cost, fast, and may be carried out using a relatively wide spectrum of hardware (e.g. commercial cameras, scanners, manual to full automated imaging systems). Therefore, even real-time (or near real-time) evaluation of a huge number of grains have been possible for various purposes in research and industry.

The techniques utilized for image-based grain analysis can be categorized under the term of high-throughput phenotyping (HTP), which has been emerged as an efficient paradigm in response to the need for keeping the feasibility of investigations in the current complex and large-scale breeding programs.

The most frequent sensors used in HTP are the efficient, inexpensive, and widely available RGB cameras [19] A simple processing of an RGB image of grains along with utilizing appropriate indices of size, color, and shape, can thoroughly and rapidly quantify the phenotype of grain samples. It seems most reasonable to select the projected area (*Area*) as the most relevant image-derived index for estimating grain weight; as this indicator provides a 2D representation of the 3D grain size (compared with the one-dimensional criteria e.g. grain width or length). Accordingly, studying the relationship between the area and weight of individual grains, Kim et al. [20] introduced a single power model equation for estimating wheat grain weight, (i.e. $weight={area}^{1.32}$), which provided a higher precision compared with the linear model.

In a preliminary analysis conducted with the aim of evaluating the variations of grain size and shape in wheat cultivar mixtures (see [21]), it was observed accidentally that grain weight had a relatively higher correlation with grain width, compared with the well-assessed index of projected grain area. This observation encouraged a more comprehensive analysis for potentially improving the image-based estimation of wheat grain weight. Therefore, the purposes of the present study were (i) assessing and documenting the relative advantage of grain width; (ii) seeking more efficient image-derived indices for predicting grain weight; and (iii) considering the technical requirements emerged during analyses, effect of image resolution enhancement on the weight prediction was also evaluated.

## Results

### Evaluation of image-derived indices

**Fig. 1:**
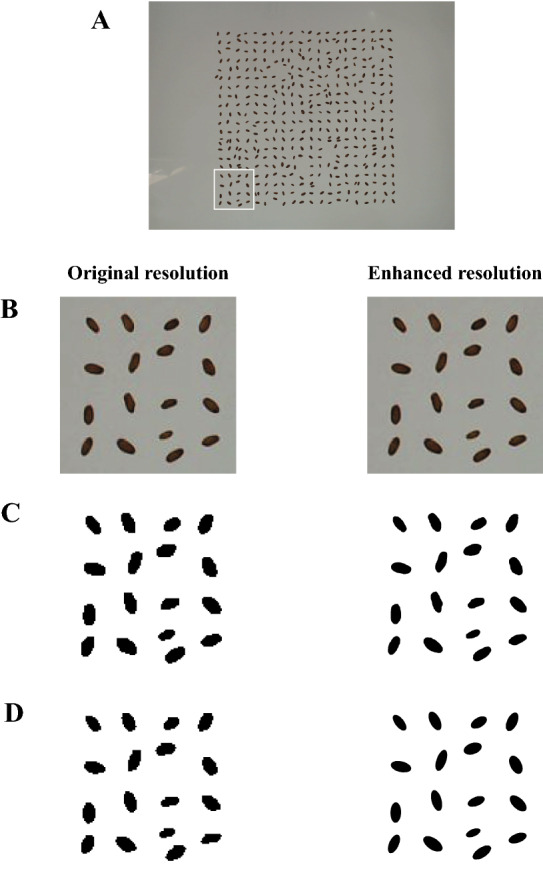
Output of image segmentation for extracting grains and fitting the best ellipses. **A** A single image from the archive with more than 400 wheat grains. As an example, the grains in the white frame are processed in the next parts of the figure. **B** Output of resolution enhancement; **C** Result of image segmentation. A same thresholding is used for both resolutions; **D** Fitting the best ellipses to the single grains

Seeking more robust image-derived indices for grain weight prediction, an image archive of wheat grains was processed, which were harvested from a 2-year field experiment carried out with 3 replicates under two irrigation conditions and included 15 cultivar mixture treatments. As shown in Fig. 1, enhancing the image resolution improved the quality of grain segmentation and ellipse fitting, considerably. This improvement was consequently reflected in the precision of the correlations and linear models developed for prediction of MGW (which will be discussed later).

**Fig. 2:**
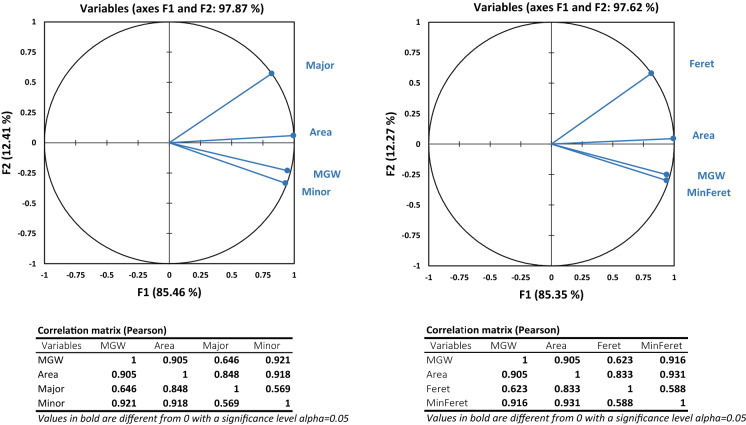
Principal Component Analysis (PCA) of mean grain weight (MGW) and basic image-derived indicators of grain size, i.e. major and Feret (indices of grain length), minor and minimum Feret (indicators of grain width), and area. Obviously, the one-dimensional indicators of grain width reflect the variations of MGW more precisely than the two-dimensional factor of area

Principal component analysis (Fig. 2) indicated that in comparison with area (R = 0.905), the grain width had a stronger relationship with MGW; regardless of which width indicator was used (R = 0.921& R = 0.916 in the cases of using *Minor* and *MinFeret*, respectively).

**Fig. 3:**
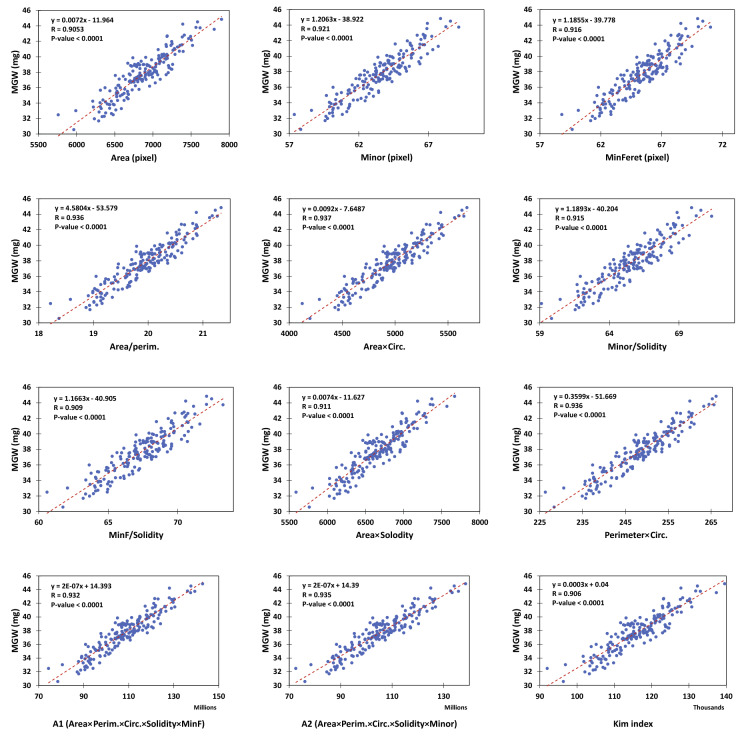
The correlations between mean grain weight (MGW) and image-derived indices. Here, the images with enhanced-resolution were used

Besides the two control indices i.e. *Area* and *Kim index*, the correlation of MGW with 33 other preliminary indices were also tested; among which 10 indices with comparatively higher correlations than the two controls were selected for further analyses (Table 1). Figure 3 shows the correlations between MGW and the selected indices derived from the enhanced resolution images. The indices of *Area* × *Circ.*, *Perim.* × *Circ.*, and *Area/Perim.* had relatively stronger relationships with MGW. Table 2 also indicates the variations in the correlation coefficients (R) in various environmental conditions. It is obvious that almost in every condition, the selected indices had a comparatively higher relationship with mean grain weight, compared with *Area* and *Kim index*. Also, the three indices mentioned before (i.e. *Area* × *Circ.*, *Perim.* × *Circ.*, and *Area/Perim*) had the highest R values, almost in every conditions. Moreover, in consistency with the fact shown in Fig. 1, the enhanced resolution improved the correlations considerably.

**Table 1:** List of the empirical image-derived indices tested in the present study

Preliminary indices	Selected indices
Area	Area
Perimeter (Perim.)	Minor
Major	MinFeret
Minor	Area/perim
Circularity (Circ.)	Area × Circ
Feret	Minor/Solidity
skewness (Skew)	MinF/Solidity
kurtosis (Kurt)	Area × Solodity
MinFeret (MinF)	Perim. × Circ
Aspect ratio (AR)	A1 (Area × Perim. × Circ. × Solidity × MinF)
Round	A2 (Area × Perim. × Circ. × Solidity × Minor)
Solidity	Kim index
Minor/Major	
MinF/Feret	
Area/MinF	
Area/Minor	
MinF/Minor	
Area/perim	
Minor/Perim	
MinF/Perim	
Area/(Perim.^2)	
MinF × Area/Perim	
Area/MinF	
Area/Minor	
Circ. × Solidity	
Area × Circ	
MinF × Circ	
MinF/Solidity	
Feret/Solidity	
Area × Solodity	
Feret × MinF × Solidity	
Perim. × Circ	
A1 (Area × Perim. × Circ. × Solidity × MinF)	
A2 (Area × Perim. × Circ. × Solidity × Minor)	
Kim index	

At the first step, the correlations between mean grain weight and the preliminary image-derived indices were tested. Then, the indices with a higher correlation coefficients (R) than those of the two control indices, i.e. "Area" and "Kim index", were selected for further analyses. Kim index (i.e. Area1.32) was derived from the study of Kim et al., 2021. For definition of the other basic indices, see the ImageJ user guide on "Analyze particles…" at https://imagej.nih.gov/ij/docs/guide/146-30.html

**Table 2:** The correlation coefficients (R) of mean grain weight (MGW) and image-derived indices

Resolution	Indices	Overall	1st year	2nd year	WI (2 years)	DI (2 years)	1st Y. WI	1st Y. DI	2nd Y. WI	2nd Y. DI
Original resolution	Area	0.8740	0.8382	0.8813	0.8092	0.8486	0.8117	0.7898	0.7805	0.8113
	Minor	0.8790	0.9044	0.9034	0.7805	0.8549	0.8751	0.8621	0.7860	0.8310
	MinFeret	0.8833	0.9030	0.9086	0.7930	0.8567	0.8664	0.8579	0.7897	0.8488
	Area/perim	0.8920	0.9034	0.9087	0.8083	0.8712	0.8737	0.8690	0.7972	0.8454
	Area × Circ	0.8921	0.9081	0.9088	0.8118	0.8688	0.8846	0.8704	0.8004	0.8467
	Minor/Solidity	0.8863	0.9080	0.9044	0.7959	0.8627	0.8759	0.8666	0.7866	0.8373
	MinF/Solidity	0.8852	0.8960	0.9062	0.8038	0.8541	0.8584	0.8425	0.7856	0.8498
	Area × Solidity	0.8776	0.8478	0.8884	0.8085	0.8534	0.8222	0.8022	0.7869	0.8194
	Perim. × Circ	0.8902	0.9088	0.9087	0.8108	0.8646	0.8906	0.8702	0.8006	0.8438
	A1 (Area × Perim. × Circ. × Solidity × MinF)	0.8942	0.8958	0.9074	0.8228	0.8706	0.8690	0.8525	0.8069	0.8515
	A2 (Area × Perim. × Circ. × Solidity × Minor)	0.8946	0.8984	0.9076	0.8222	0.8714	0.8755	0.8546	0.8078	0.8498
	Kim index	0.8743	0.8390	0.8815	0.8104	0.8489	0.8130	0.7902	0.7810	0.8129
	Mean	0.8852	0.8876	0.9013	0.8064	0.8605	0.8597	0.8440	0.7924	0.8373
Enhanced resolution	Area	0.9053	0.8688	0.8963	0.8617	0.8812	0.8468	0.8204	0.8163	0.8108
	Minor	0.9208	0.9433	0.9257	0.8582	0.9069	0.9077	0.9255	0.8524	0.8434
	MinFeret	0.9159	0.9367	0.9204	0.8456	0.9032	0.8878	0.9241	0.8416	0.8344
	Area/perim	0.9361	0.9421	0.9314	0.8889	0.9225	0.9115	0.9236	0.8642	0.8588
	Area × Circ	0.9373	0.9463	0.9334	0.8922	0.9228	0.9194	0.9280	0.8714	0.8615
	Minor/Solidity	0.9149	0.9389	0.9150	0.8471	0.8991	0.8994	0.9212	0.8287	0.8261
	MinF/Solidity	0.9088	0.9307	0.9084	0.8328	0.8935	0.8777	0.9171	0.8161	0.8148
	Area × Solidity	0.9110	0.8754	0.9052	0.8674	0.8882	0.8527	0.8290	0.8297	0.8242
	Perim. × Circ	0.9362	0.9485	0.9335	0.8932	0.9198	0.9246	0.9304	0.8782	0.8582
	A1 (Area × Perim. × Circ. × Solidity × MinF)	0.9323	0.9292	0.9279	0.8853	0.9170	0.8978	0.9057	0.8622	0.8568
	A2 (Area × Perim. × Circ. × Solidity × Minor)	0.9353	0.9338	0.9310	0.8910	0.9201	0.9058	0.9106	0.8691	0.8614
	Kim index	0.9055	0.8690	0.8969	0.8618	0.8819	0.8471	0.8208	0.8159	0.8129
	Mean	0.9216	0.9219	0.9188	0.8688	0.9047	0.8898	0.8964	0.8455	0.8386

WI and DI: well- and deficit-irrigated, respectively

### Effect of treatments on the indices

Analysis of variance (ANOVA; Table 5) also indicated that the effects of year, mixture treatments, and water stress were very significant on MGW, as well as the two control and 10 selected indices (data not shown; P < 0.0001). As it was expected according to the high correlations between MGW and the image-derived indices, the variation of the indices followed completely the changes in MGW; i.e. the post-anthesis water stress reduced the values significantly (e.g. MGW reduced from 39.291 mg under well-irrigation to 36.157 mg under deficit-irrigation conditions, averaged between 2 years; data not shown). In average, MGW also reduced significantly from 39.264 mg in the 1st season to 36.184 mg in the 2nd season (noteworthy, the effect of season on grain yield and most agronomic features were significant. For more information, see [21]). All of the 12 indices showed a similar trend. As a whole, values of MGW and the correlated visual indices were lower in the higher yielding treatments (or conditions) and vice versa; mainly due to the strong negative relationship between grains m^−2^ and MGW on one hand, and the high correlation between grain yield and grains m^−2^ at the other hand (see [21]). The main implication of this observation for the present study was that the variations of the visual indices were highly consistent with those of MGW; regardless of the sources of variation, i.e. significantly different growing seasons, water stress, or mixture treatments.

### Model validation

**Fig. 4:**
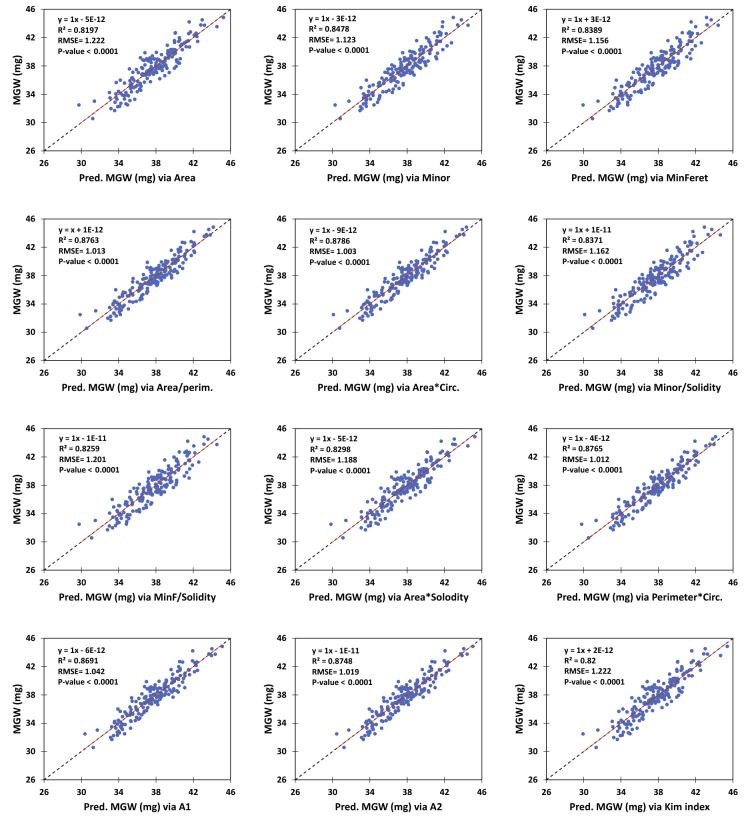
Performance of linear models developed for predicting mean grain weight (MGW) using the superior image-derived indices**.** The red and dashed lines show the linear trend and 1:1 line, respectively

Figure 4 represents the performance of the linear models developed using the selected indices for predicting MGW (here the images with enhanced resolution were used). As it was expected based on the previous results, all of the ten linear models predicted MGW with a more accuracy compared with the two control indices (RMSE values ranged between 1.003 to 1.201, for the *Area* × *Circ.* and *MinFeret/Solid.* models, respectively; Fig. 4). Results of cross-validation and also model parameters have been shown in Table 3. As expected, root mean square errors of cross-validation, followed the pattern of RMSEs reported earlier, i.e. errors of *Area* × *Circ.* < *Perim.* × *Circ.* < *Area/Perim*. Table 3 also represents the reduction percentages of RMSE due to the enhanced resolution by the factor of 10. As a whole, the effect of resolution enhancement was more considerable on the precision of the indices which were based on shape properties (e.g. the products of *circularity*), rather than the size-based features (*Area,* or *MinFeret*).

**Table 3:** Cross-validation and parameters of the linear models developed for estimation of mean grain weight (MGW; mg) using image-derived indices

Indices	Original resolution (pixel)				Enhanced resolution (pixel)				SI (mm/ or mm2)				Cross-validation		RES-based RMSE improvement (%)
	Slope	Slope	Slope	Slope	Slope	Intercept	R^2^	RMSE	Slope	Intercept	R^2^	RMSE	Overall RMSE	Micro average RMSE	
Area	0.6876076	–14.13069	0.7639	1.39916	0.007242	–11.96377	0.8197	1.22279	3.4546529	–10.50562	0.8197	1.22279	1.218 ± 0.165	1.228 ± 0.000	12.61
Minor	11.473872	–40.04071	0.7726	1.37308	1.206266	–38.92239	0.8478	1.12329	26.223532	–37.43548	0.8501	1.11472	1.117 ± 0.121	1.123 ± 0.000	18.19
MinFeret	11.479164	–47.28420	0.7802	1.34983	1.185451	–39.77784	0.8389	1.15583	25.751014	–38.21530	0.8409	1.14849	1.150 ± 0.134	1.157 ± 0.000	14.37
Area/perim	43.088447	–56.75704	0.7957	1.30144	4.580449	–53.57889	0.8763	1.01287	98.930057	–51.22634	0.8759	1.01427	1.011 ± 0.143	1.020 ± 0.000	22.17
Area × Circ	0.7810103	–9.19948	0.7958	1.30124	0.009157	–7.648741	0.8786	1.00327	4.3865946	–6.502854	0.8784	1.00403	1.001 ± 0.135	1.009 ± 0.000	22.90
Minor/Solidity	11.300208	–47.78814	0.7856	1.33331	1.189310	–40.20437	0.8371	1.16220	25.845173	–38.66377	0.8396	1.15317	1.157 ± 0.126	1.163 ± 0.000	12.83
MinF/Solidity	11.243958	–55.24627	0.7836	1.33950	1.166277	–40.90504	0.8259	1.20137	25.326662	–39.29586	0.8282	1.19332	1.196 ± 0.139	1.203 ± 0.000	10.31
Area × Solidity	0.7367298	–12.03813	0.7702	1.38042	0.007417	–11.62672	0.8298	1.18779	3.5390870	–10.18897	0.8295	1.18879	1.183 ± 0.158	1.193 ± 0.000	13.95
Perim. × Circ	3.3790373	–54.79264	0.7925	1.31155	0.359924	–51.66935	0.8765	1.01190	7.7804528	–49.44107	0.8764	1.01215	1.011 ± 0.121	1.017 ± 0.000	22.85
A1 (Area × Perim. × Circ. × Solidity × MinF)	0.0017579	13.56291	0.7996	1.28896	2.1504E-07	14.393318	0.8691	1.04158	0.0505571	15.011033	0.8685	1.04405	1.038 ± 0.149	1.047 ± 0.000	19.19
A2 (Area × Perim. × Circ. × Solidity × Minor)	0.0018807	14.06366	0.8003	1.28685	2.2128E-07	14.390431	0.8748	1.01874	0.0520301	15.005713	0.8742	1.02147	1.016 ± 0.143	1.025 ± 0.000	20.83
Kim index	0.1306980	–1.60504	0.7644	1.39762	0.000325	0.0399900	0.8200	1.22159	1.1264207	1.1437907	0.8200	1.22156	1.216 ± 0.164	1.226 ± 0.000	12.59

MinF, Perim., and Circ. are minimum Feret diameter, perimeter, and circularity, respectively.

Original resolution, enhanced resolution, and also SI are the various scales of the image dimension based on which the analyses have been carried out.

The output slopes and intercepts of cross-validation were exactly the same as the SI parameters (noteworthy, the cross-validation was conducted using all of the 180 observations).

The last column (Resolution-based RMSE improvement), indicates the percentage of reductions in RMSE of grain weight prediction due to the resolution enhancement

### Further evidence and implications for the role of grain width

For better understanding of the relationship between the best predictive indices and the basic grain shape parameters, additional correlations were also conducted. In this evaluation, the data of all single grains (i.e. 19,596 grains) of monocultures were used, and *Major* and *Minor* were chosen as the measurers of the grain length and width, respectively. As shown in Table 4, it was found that:

(i) The superior indices had the highest correlations with *Minor* (grain width), rather than with their mathematical components. For instance, see the correlation between *Area/Perim.* and *Minor* (R = 0.987) vs. the correlation between the *Area/Perim.* ratio and the relevant parameters i.e. *Area* (R = 0.973) and *Perimeter* (R = 0.875; Table 4).
(ii) Among the main grain dimensions, *Area* had the highest correlation with *Minor* (grain width), while *Perimeter* depended most on *Major* (grain length).
(iii) The correlation between the two main grain axes, i.e. *Major* and *Minor*, was not such high (R = 0.608) that one could be estimated precisely based on the other. It implies a relatively independence between the grain growth (and/or filling) along the length and width directions.

**Table 4:** The coefficients of correlation (R) among the basic shape factors and the three superior synthetized indices used for mean grain weight prediction

Parameters	Major	Minor	Area	Ellipse area	Perim	Area/Perim	Area × Circ
Minor	0.608						
Area	0.849	**0.932**					
Ellipse area	0.849	**0.932**	1				
Perim	**0.959**	0.801	0.958	0.958			
Area/Perim	0.719	**0.987**	0.973	0.973	0.875		
Area × Circ	0.717	**0.984**	0.975	0.975	0.873	0.997	
Perim. × Circ	0.719	**0.987**	0.973	0.973	0.875	1	0.997

In this analysis, data of 19,596 grains sampled from the monocultures of 4 early- to middle-ripening cultivars was used (enhanced-resolution images were processed).

All correlations were very significant (P < 0.0001).

The bolded values show the superior correlation of basic shape factors (i.e. *Major*, *Minor*, *Area*, *Ellipse area*, or *Perimeter*) in each row.

"*Ellipse area*" is the area of the best ellipse fitted on the grain, and calculated as follows (in the present evaluation, the difference between *Area* and *Ellipse area* was almost zero, i.e. in average less than % 2.3 × 10^–8^): ${Ellipse\, area}=(Major/2)\times (Minor/2)\times \pi$

**Fig. 5:**
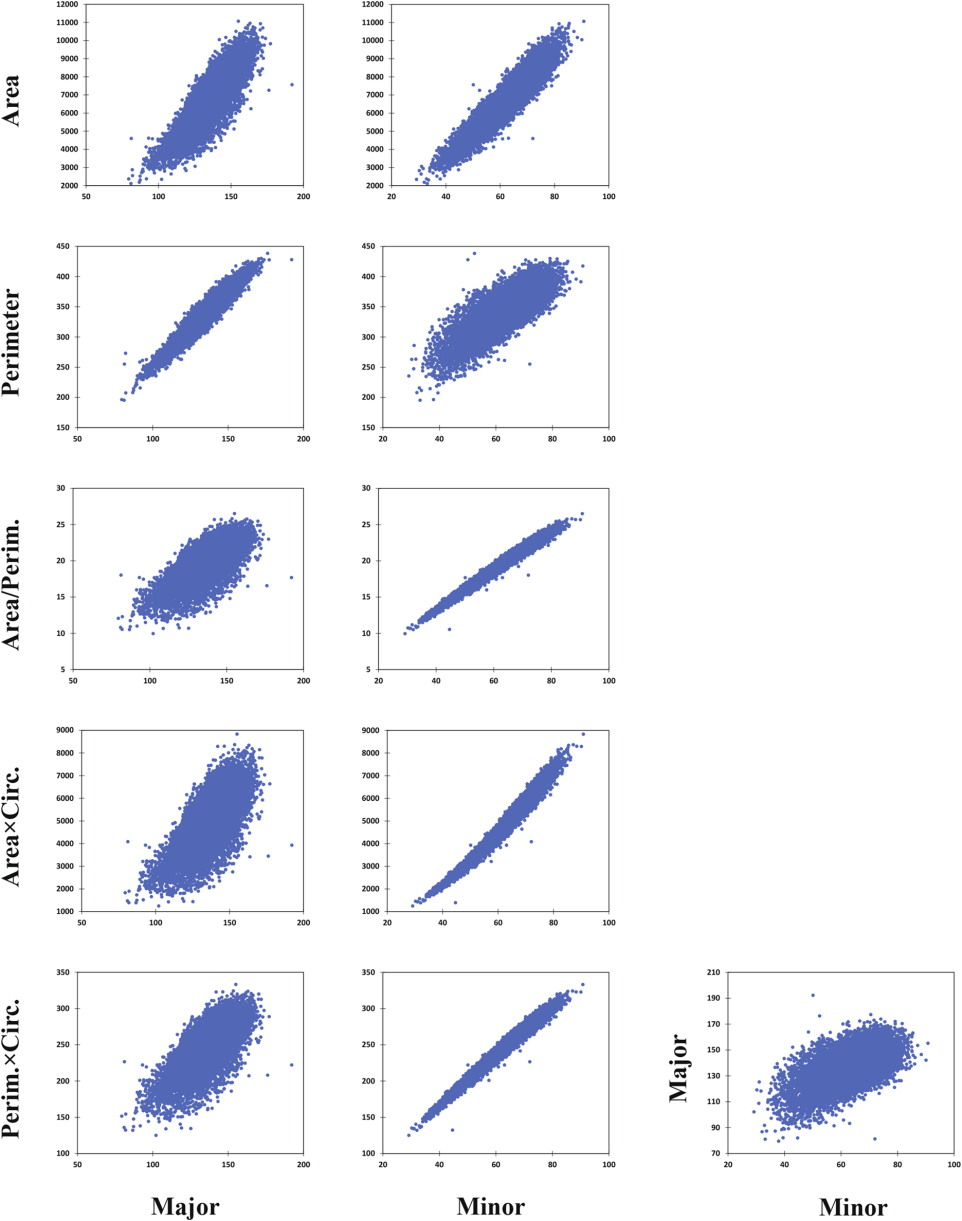
The correlations among *Major* and *Minor* (representatives of grain length and width, respectively) and other basic shape factors, and also superior synthetized weight indicators. *Major* and *Minor* are the largest and shortest axes of the best ellipse fitted on each grain. Unit of all dimensions is pixel (the enhanced-resolution images of 19,596 grains sampled from all monocultures of early- to middle ripening cultivars grown during two seasons under well- and deficit-irrigation were used). For more details and coefficients see Table 4

These findings have been also represented in Fig. 5, which indicates the comparative correlations of *Major* and *Minor* with the basic shape features and superior predictive indices. Besides, Supplementary file 1 provides more detailed information and graphs of the respective correlations as affected by various treatments of irrigation and cultivars. As a whole, the trends described above remained almost consistent across different irrigation conditions and/or cultivars with dissimilar ripening dates; in spite of that the effects of season, irrigation treatment, and cultivars on the grain length and width were very significant (Table 5). Besides, as indicted in Table 5, the significantly different classes of MGW and *Minor* in various cultivars were exactly the same.

**Table 5:** Effects of year, irrigation, and cultivar on Major and Minor (i.e. the measurers of grain length and width, respectively) and mean grain weight of monocultures

Ellipse axis	Year				Irrigation				Cultivar (monocultures)										C.V. (%) of single grains **
	1st	2nd	% Difference	P-value	WI	DI	% Difference	P-value	1st		2nd		3rd		4th		Range (%)	P-value *	
Major (mm)	6.237	6.086	2.480	** < 0.0001**	6.205	6.119	1.377	** < 0.0001**	5.942	d	6.190	b	6.415	a	6.101	c	7.680	** < 0.0001**	**8.580**
Minor (mm)	2.897	2.852	1.566	**0.0015**	2.950	2.799	5.127	** < 0.0001**	2.832	c	2.985	a	2.882	b	2.798	c	6.508	** < 0.0001**	**12.362**
MGW (mg)	39.139	36.566	6.574	** < 0.0001**	39.561	36.143	8.640	** < 0.0001**	36.58	c	40.43	a	38.32	b	36.08	c	11.505	** < 0.0001**	–

All comparisons except for the last column (C.V.) have been carried out using the mean values of images (i.e. only the 48 observation belonged to the monocultures)

Bolded values show the significant differences (P < 0.001).

*MGW* mean grain weight, *WI* well-irrigated, *DI*: deficit-irrigated

^*^Tukey test; different letters in each row, show the significant differences among cultivars.

**Coefficient of variation calculated only using the data of single grains of monocultures (including 19,595 grains)

## Discussion

The idea of the present study was exploring more efficient visual indices for wheat MGW prediction, other than 2D grain area. For this purpose, various empirical indices of grain size and shape were evaluated using image processing. It was observed that among the size criteria, the one-dimensional indices of grain width (i.e. *Minor* and *MinFeret*) had relatively higher correlations with MGW, compared with the two-dimensional index of grain area, or perimeter (the latter of which was filtered out in the preliminary assessments; R = 0.801 when the enhanced-resolution images were used, data not shown). This observation inspired that there might be also other unexplored indices for MGW, which originate from the exclusive physiology of wheat crop, e.g. the processes associated with the grain filling capacity. Therefore, the correlation of MGW with some of the conventional shape indices and also several empirical criteria were tested.

*Area* × *Circ.*, *Perim.* × *Circ.*, and *Area/Perim.* were the superior indices in prediction of MGW using the linear models, and indicated a relatively consistent performance across the various conditions. Furthermore, almost under every of the 4 environmental conditions, other selected indices could predict MGW with a higher precision compared with area. Besides the applicable aspect of this finding, it is also an evidence for the possibility of improving wheat grain weight estimation by exploring new visual indicators. Based on the formula of the *circularity* index used in ImageJ (see https://imagej.nih.gov/ij/docs/guide/146-30.html), all of the three superior indices have a common factor i.e. the *Area/Perim.* ratio:

1 $$Circularity=\frac{4\pi \times Area}{{Perimeter}^{2}}$$

$$Therefore:$$

2 $$Area\times Circularity=Area\times \left(\frac{4\pi \times Area}{{Perimeter}^{2}}\right)=4\pi \times {\left(\frac{Area}{Perimeter}\right)}^{2}$$

3 $$Perimeter\times Circularity=Perimeter\times \left(\frac{4\pi \times Area}{{Perimeter}^{2}}\right)=4\pi \left(\frac{Area}{Perimeter}\right)$$

So, the formulae of the two other indices (i.e. *Area* × *Circ.*& *Perim.* × *Circ.*) might be slightly simplified, and consequently the computational cost could be reduced. Such conversions may be particularly important in high-throughput phenotyping; where a considerable number of grains should be analyzed in real-time e.g. using high-speed imaging systems. Besides, these observations imply that the majority of the efficient indices evaluated in the present study are based on two fundamental factors: (i) grain width (measured by *Minor* & *MinFeret*), and (ii) the *Area/Perim.* ratio. Of course, additional correlation tests indicated that the *Area/Perim.* ratio, as the same as other superior indices, had in turn correlated strongly with grain width.

As described before, enhancing the image resolution by the factor of 10 improved the predictive precision of the indices considerably. However, this improvement was not equal for all of the selected indices; as those which were independent of the grain shape, were less influenced (e.g. the size indicators such as *Area* or *MinFeret*; see Table 3). In contrast, the shape-depended indices showed considerably higher degrees of improvement in MGW prediction (for instance, see the indices with the factor of *Circularity*, or even *Minor*, which is resulted from ellipse fitting; see Fig. 1). Therefore, it is necessary to ensure the desirable image resolutions (which is achievable either at the time of imaging/scanning, or using interpolation), before running the analyses.

Noteworthy, since in the present study the weight analysis was designed and carried out based on the average values, generalization of the findings and models for estimating weight of individual grains might require further assessments. However, considering that each of the 180 samples was consisted of more than 400 grains, it is expected that both types of estimations (i.e. MGW and individual grain weight) should be highly correlated. As an evidence for this fact, it was observed that similar to the study of Kim et al. [20], *Kim index* provided a more precise grain weight estimation than *Area*. More importantly, slopes of the corresponding linear models calculated in both studies were almost similar (see Table 3); despite the differences in the genotypes, treatments, imaging systems, lighting, and probably the image processing algorithms:

$$\mathrm{Kim\, et\, al.}: \left\{\begin{array}{l}Weight=\left(3.46\times Area\right)-15.99 \\ Weight=\left(27.02\times Width\right)-50.48\end{array}\right.$$

$$\mathrm{Present\, study}: \left\{\begin{array}{l}Weight=\left(3.45\times Area\right)-10.50 \\ Weight=\left(25.75\times MinFeret\right)-38.21\\ Weight=\left(26.22\times Minor\right)-37.43\end{array}\right.$$

(units: mg, mm^2^, and mm).

Besides the technical advantageous for developing phenotyping platforms, findings of the present study might also be readily used in wheat physiology and breeding approaches. For instance, the relatively stronger relationship between MGW and grain width (vs. length or even area) may provide valuable implications for the grain development and/or filling processes; particularly despite the fact that (i) grain filling is an acropetal process and mainly occurs in the grain length direction, and (ii) the 2D grain area provides the information of 2 out of the 3 dimensions (so theoretically, it is expected to be a more significant weight contributor compared with the one dimensional traits such as grain width). Moreover, it was evidenced that the superior predictive indices had the highest correlations with grain width, which had even exceeded the same correlations of the indices with their own mathematical components (see Table 4, Fig. 5, and Additional file 1). Therefore, having a frequent and prominent appearance in the present study, grain width seems to be a fundamental and unique trait in grain physiology and weight assessments. The results also seem to be consistent with the findings of Gegas et al. [9] who provided the genetic evidences for an emerging phenotypic model where wheat domestication has transformed a long thin primitive grain to a wider and shorter modern grain. In addition, comparative variations and contribution of the two main axes to grain weight may open new window into the grain development assessments and yield physiology. Indeed, grain length and width might be supposed as the components of weight, or in a more general view, as the subcomponents of wheat grain yield. Conducting sufficient researches, such framework could provide valuable information about the pattern of grain development or filling in the main perpendicular dimensions, particularly under various conditions; e.g. in the present study, post-anthesis water stress (50% of filed capacity) reduced the grain length and width significantly by 1.38% & 5.13%, respectively, in monocultures; which overall led to 8.64% reduction in MGW (Table 5). This suggests that the water stress treatment had affected the grain extension (the interaction of development and filling) along the width direction more considerably than along the grain length. In contrast, the effect of growing season on the grain length was higher than on the grain width (i.e. reduced the respective values in the second year by 2.48% vs. 1.57%, respectively; which resulted in 6.57% MGW reduction). Therefore, it can be concluded that the season had more affected the earlier developmental grain phases (in which the potential of final length is determined), while the post-anthesis water stress had influenced –more considerably- the later phenological stages and filling period (which contributes more to grain width). In a similar way, various pheno-physiological aspects of genetic or environmental effects on the wheat grain might be evaluated more finely in a subcomponent level of grain yield.

In addition to the main applications of the findings reported here, (i.e. grain weight predictions or physiological assessments), the image-derived indices could be used for automated seed screening and grain sorting purposes; e.g. the less-matured grains might be easily detected and filtered out by appropriate thresholding of grain dimensions or predictive indices. Determining the best quantitative thresholds requires further studies. Also, the superior visual indices introduced in the present study might be used as the selection criteria in breeding programs (e.g. see [6]); before which the efficiency and stability of the indices should be tested using a more heterogeneous collection of genotypes grown under a broader environmental conditions. In general, the image-based MGW predictive method reported here, along with the other related applications could increase the speed, accuracy, and frequency (i.e. replication) of crop sampling and grain assessments; which in turn, might reduce the experimental error and improve the agro-physiological evaluations.

## Conclusion

The present study was conducted to explore more efficient image-derived indices for predicting wheat MGW. For this purpose, simple size and shape indices of cultivar mixtures grown under 4 environmental conditions (2 seasons × 2 water conditions) were analyzed. It was observed that MGW had a higher correlation with 10 out of the more than 30 evaluated empirical indices, compared with the well-assessed indicators of projected area (i.e. *Area* & *Kim index*). The best MGW predictions were obtained using the *Area* × *Circ.*, *Perim.* × *Circ.*, and *Area/Perimeter* indices. In general, the majority of the superior indices had one of the two common factors in their structure, i.e. either were based on grain width (evidenced as *Minor* & *MinFeret*) or the *Area/Perimeter* ratio; the latter of which had in turn high correlation with the first. Therefore, having a prominent appearance in the present study, grain width was introduced as a fundamental predictive index for weight estimations. The comparative precision of the ten selected indices was stable under different environmental conditions. Moreover, it was observed that enhancing the image resolution by the factor of 10 could considerably improve the MGW predictions; particularly when the shape-based indices were used. In conclusion, it is expected that utilizing the simple predictive linear models developed and validated using the superior image-derived indices, particularly grain width, could increase the precision of MGW estimations, and also facilitate wheat physiological assessments.

## Methods

### Field experiment

In order to explore new image-derived indices to improve prediction of wheat grain weight, an archive of images taken from the harvested grains of a 2-year field study was analyzed. The goal of the field experiment was studying the responses of wheat cultivar mixtures with various ripening patterns to normal and post-anthesis water stress conditions (see [21]); which was conducted during 2014-15 and 2015-16 growing seasons at the research field of the School of Agriculture, Shiraz University, Iran (29°73´ N latitude and 52°59´ E longitude at an altitude of 1,810 masl). Mixture treatments were 15 mixing ratios of four early- to middle-ripening wheat cultivars (Chamran, Sirvan, Pishtaz, and Shiraz, respectively) including the 4 monocultures and their every 11 possible mixtures, which were grown with 3 replicates under two well-irrigation and post-anthesis deficit-irrigation conditions. The experimental design was RCBD (Randomized Complete Block Design) in which all the 90 (2×2 meter) plots were arranged in a lattice configuration with 1 meter distances. Plant density was 450 plants/m^2^ and seeds were mixed in each year with equal ratios (i.e. 1:1, 1:1:1, and 1:1:1:1 for the 2-, 3-, and 4-component blends, respectively), considering their 1000-grain weights and germination percentages. The planting date in the first and second growing seasons were November 20 and November 5, respectively; and based on the soil test, 150 kg nitrogen/ha was applied (as urea) in three equal splits i.e. at planting, early tillering, and anthesis. No pesticide was used and weeding was done by hand once at stem elongation.

Irrigation interval was 10 days based on local practices, and the amount of irrigation water was estimated using the Fao-56 Penman–Monteith model with local corrected coefficients which was reduced to 50% of evapo-transpirational demand from the first irrigation after anthesis. Late in the season, plants were harvested from the center of plots and yield components were estimated using a laboratory thresher and weighing.

### Imaging

Images were taken from the archive of an exclusively designed laboratory system (Visual Grain Analyzer, VGA), which was equipped with a Logitech HD Pro Webcam C920 mounted on an adjustable arm, a glass table with a 60 × 60 cm flicker-free white LED panel beneath it as the light source, and a professional software written in C# for real-time screening of the grains. Imaging was carried out for other purposes, so the properties were not necessarily designed for the present study. Accordingly, images were taken under ambient light from 43.5 cm above the samples (i.e. lens to the table), and the image dimensions were 960 × 720 pixels (i.e. the original resolution was ≈ 7 MP). For each experimental plot, more than 400 grains were sampled randomly and arranged on the imaging table using a Vacuum Seed Counter, so that there was no contact between the grains. Therefore, the total dataset (including 90 images for each year) was consisted of the data of more than 72,000 single grains. Immediately after imaging, the grains of each image were weighed using a A&D EK-610i (d = 0.01 g) weighing balance. Mean grain weights were calculated by dividing the sample weight by the number of grains.

### Image processing

Since the VGA system has not been commercialized or released yet, and also the analyses had to be kept reproducible, only the data of grain size (for conversion of pixel to mm) was taken from this system; and all of the image analyses were carried out using ImageJ version. 2.1.0/1.53c [22]. First, the grains were segmented from the background using the *Color thresholding* tool (Image > Adjust > Color thresholding). The thresholding method and color space were set as “Default” and HSB, respectively. Thereafter, size and shape features of grains were calculated using the *Analyze particles* tool. For this purpose, the attended features were selected in the *Set Measurements* menu (*Analyze* > *Set Measurement*), and *Analyze Particles* was run. Before running, the “*Show Ellipses*” option was selected, and no size or circularity filtering was applied on the sample. The output tables were saved as.csv files and used for next analysis. As described before, it was found that enhancing the image resolution could improve the estimations. Therefore, in another analyses, before running the “*Analyze Particles*”, the resolution of images was enhanced using the Bicubic algorithm and by factor of 10 (i.e. both image dimensions were multiplied by 10, so the image resolution was increased 100 times). Resizing the images was carried out using the *Batch* processing tool (Process > Batch > Convert; and *interpolation* and *scale factor* were set to Bicubic & 10, respectively).

**Fig. 6:**
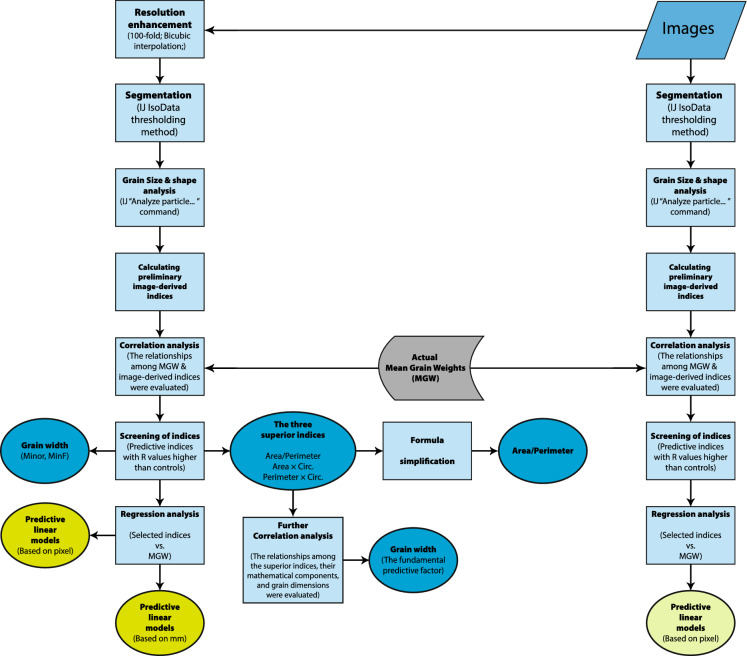
The pipeline of image processing and analyses carried out in the present study. *IJ* ImageJ, *MGW* mean grain weight, *R* the correlation coefficient

Using the output of image processing, the averaged values of basic features of size and shape were calculated for each image, and the correlation of these visual indices with MGW were evaluated. The examples of basic indices included *area*, *perimeter*, the major and minor axes of the best fitted ellipses to the grains (*Major* & *Minor*; also see [4]), minimum (*MinFeret*) and maximum (*Feret*) caliper diameter, *Circularity* (a value between 0 to 1 for an infinitely elongated shape to a perfect circle), *solidity* (the ratio of area to the convex hull area), etc. Besides the basic features, the correlation of MGW with several synthesized indices were also tested; which were the products or ratios of the basic indices. *A*_*1*_ and *A*_*2*_ were among the instances of synthesized indices which are the products of the 5 most efficient basic indices. The full list of the evaluated indices is represented in Table 1. Also for more detail of the definitions and formulae, see https://imagej.nih.gov/ij/docs/guide/146-30.html. Linear correlations of MGW with the visual indices were compared with those of the two control criteria i.e. $Area$ and *Kim index* (${Area}^{1.32}$; taken from the paper of Kim et al. [20]), and the indices with a higher correlations than the controls were selected as the final indicators of MGW. Using each of the selected indices, a linear model for prediction of MGW was developed and evaluated. Although the analyses were based on the number of pixels (as the unit of dimension), in order to generalize the model parameters, outputs were also converted into mm using the data of VGA system. Moreover, ten-fold cross-validation (K = 10) was used in Rapidminer (Version 9.9) to validate the results of datamining models, in which the default values and settings of the software were chosen. All other analyses, including correlating, Principal Component Analysis (PCA), and fitting the linear models were carried out using XLSTAT (Version 2016.02.28451; Addinsoft). Figure 6 represents the pipeline of image processing and analyses carried out in the present study. Noteworthy, the image archive used in this research (with the original resolution) along with the mean values of extracted quantities have been shared on Figshare, at [23]: https://figshare.com/articles/dataset/Images_of_wheat_grains/18480722. Moreover, the image processing and calculations reported here can be simulated using a user-friendly ImageJ macro (Visual Grain Analyzer, VGA v. 1.0.1), which has been shared on GitHub at : https://github.com/haqueshenas/Visual-Grain-Analyzer. Running the code with the default settings will reproduce the reported results.

## Supplementary Information

**Additional file 1.** Results of the correlation tests among the basic grain shape factors and the superior weight predictive indices.

## Data Availability

Not applicable.
